# A prospective cohort study on post COVID syndrome from a tertiary care centre in Sri Lanka

**DOI:** 10.1038/s41598-023-42350-4

**Published:** 2023-09-20

**Authors:** M. M. P. T. Jayasekera, N. L. De Silva, E. M. D. T. Edirisinghe, T. Samarawickrama, S. W. D. R. C. Sirimanna, B. G. D. S. Govindapala, G. Senanayake, D. L. N. Wickramaratne, K. Hettigoda, U. D. I. B. Gunawaradana, K. D. P. B. Wijayananda, R. A. N. K. Wijesinghe

**Affiliations:** 1 Department of Medicine, Faculty of Medicine, General Sir John Kotelawala Defence University, Colombo, Sri Lanka; 2 University Hospital Kotelawala Defence University, Colombo, Sri Lanka; 3 Department of Psychology, University of Peradeniya, Peradeniya, Sri Lanka

**Keywords:** Microbiology, Diseases

## Abstract

There is a scarcity of follow-up data on post-COVID syndrome and its physical, psychological, and quality of life attributes, particularly from South Asian populations. This study was conducted to assess the prevalence, associations, and impact of the post-COVID syndrome among patients treated at a dedicated COVID-19 treatment unit. A prospective cohort study was conducted to follow-up patients with moderate to severe disease or mild disease with co-morbidities at 2 and 6 weeks, 3 and 6 months and 1 year from discharge. Clinical notes, an interviewer-administered questionnaire and six-item cognitive impairment, Montreal Cognitive Assessment, Fatigue (11-item Chalder) and EQ5D5L questionnaires were used for data collection. All patients had follow-up echocardiograms and symptomatic patients had biochemical and haematological investigations, chest x-rays, high-resolution computed tomography of chest and lung function tests. Among 153 patients {mean age 57.2 ± 16.3 years (83 (54.2% males)}, 92 (60.1%) got the severe disease. At least a single post-COVID symptom was reported by 119 (77.3%), 92 (60.1%), 54 (35.3%) and 25 (16.3%) at 6 weeks, 3 months, 6 months and 1 year respectively. Post-COVID symptoms were significantly associated with disease severity (*p* = 0.004). Fatigue was found in 139 (90.3%), 97 (63.4%) and 66 (43.1%) patients at 2, 6 and 12 weeks respectively. Dyspnoea {OR 1.136 (CI 95% 0.525–2.455)}, arthralgia {OR 1.83(CI 95% 0.96–3.503)} and unsteadiness {OR 1.34 (CI 95% 0.607–2.957)}were strongly associated with age above 60 years. Both genders were equally affected. In multivariable logistic regression, fatigue and anxiety/depression were associated with poor quality of life (QoL) (*p* = 0.014, *p* ≤ 0.001) in 6 weeks. In cardiac assessments, diastolic dysfunction (DD) was detected in 110 (72%) patients at 2 weeks and this number reduced to 64 (41.8%) at 12 weeks. The decline in diastolic dysfunction in elderly patients was significantly higher compared to young patients (*p* = 0.012). Most post-COVID symptoms, QoL and cognition improve during the first few months. The severity of the disease and older age are associated with post-COVID symptoms. Transient DD may contribute to cardiac symptoms of post-COVID syndrome, especially in elderly patients.

## Introduction

Post-Covid Syndrome is defined as “signs and symptoms that develop during or after an infection consistent with COVID-19 which continue for more than 12 weeks and are not explained by an alternative diagnosis” by the National Institute for Health and Care Excellence (NICE) the Scottish Intercollegiate Guidelines Network (SIGN), and the Royal College of General Practitioners. Although respiratory infection is the primary clinical manifestation, COVID-19 is considered a multi-organ systemic disease that includes the lungs, heart, vascular system, brain, and other organ systems^1^.

Evidence of post-COVID sequelae has been published in several studies^1–4^. Symptoms and symptom clusters were described as owing to both physical as well as psychological changes in those studies. Most studies lacked investigations to support the stated physical symptoms. Especially, detailed assessments of cardiac functions, lung functions and radiological correlation are worth the attention considering the symptomatology. Psychological symptoms such as mood disorders, fatigue, and perceived cognitive impairment that resulted in severe negative impacts on the resumption of functional and occupational activities in patients experiencing prolonged effects after COVID-19 infection have been noted^3^. Planning long-term medical care for patients after recovering from COVID-19 infections needs to focus on all aspects of medical facilities, in order to improve the quality of life of such patients addressing the need for treatments as well as rehabilitation.

There are a few reports on the overall effects of Post-COVID syndrome from Sri Lanka and other South Asian settings. However, studies focusing on cardiac function, lung function, radiological assessment as well as psychological outcome are sparse in Asian and Sri Lankan settings. Due to geographical and cultural variations, as well as differences in health systems, manifestations, associations, and the impact of these sequelae might vary from what is reported in the West. Therefore, there is an urgent need for research on the long-term prognosis of those who have recovered from an acute COVID-19 infection in a Sri Lankan setting.

Therefore, this study was conducted to assess the prevalence, associations, and impact of post-COIVD syndrome on their wellbeing among patients treated at a selective tertiary care hospital.

## Methods

This single-centre prospective cohort study was conducted in the COVID-19 treatment unit, at the University Hospital, Kotelawala Defence University of Sri Lanka (UHKDU). All the patients admitted to the above unit UHKDU in July 2021 were consecutively screened for eligibility upon admission by assessing inclusion and exclusion criteria by a research assistant. Patients above the age of 18 years, with confirmed (SARS CoV 2 PCR positive on nasopharyngeal swab) COVID-19 with moderate to severe disease or mild disease with any co-morbidities were included in the study. We excluded pregnant women, patients transferred from other hospitals directly for ICU care, and later de-escalated to the ward setting and patients who died during the hospital stay.

Doubts about the eligibility were verified by the senior investigators (physicians). Eligible patients were invited to take part in the study and an information sheet was provided. Informed consent was obtained from the patients or legally acceptable guardians (LAG) of ill patients and consent was obtained after the recovery from the acute stage.

Baseline assessment was started at the ward before discharge and follow-up visits were planned in 2 weeks, 6 weeks, 3 months, 6 months and 1 year with clinical assessment, haematological and biochemical investigations, and radiological investigations whenever necessary.

Ethics clearance was obtained from the Ethics Review Committee, General Sir John Kotelawala Defence University, Ratmalana, Sri Lanka (RP/2021/26) and institutional approval was obtained from the University Hospital KDU. All methods were carried out in accordance with relevant guidelines and regulations by the Ethics Review Committee.

### Research tools and data collection technique

The presence of comorbidities, the severity of the illness (moderate to severe), and the results of biochemical, haematological and radiological investigations were obtained by referring to the medical records.

An interviewer-administered questionnaire was used to record symptoms at each visit.

As per the national recommendations, a patient was considered fully vaccinated if the individual has received two doses of any preparation of COVID vaccines. Definition of severity considered as asymptomatic in patients who have no symptoms that are consistent with COIVD-19, mild illness, who have any of the various signs and symptoms of COIVD-19 but did not have shortness of breath or abnormal chest imaging, moderate illness who show evidence of lower respiratory disease during clinical assessment or imaging and oxygen saturation SpO_2_ ≥ 94% on room air at sea level and severe illness, individuals who have SpO_2_ < 94% on room air at sea level, a ratio of arterial partial pressure of oxygen to fraction on inspired oxygen (PaO_2_/FiO_2_) < 300 mm Hg, a respiratory rate > 30 breaths/min, or lung infiltrates > 50% and critical illness in individuals who have respiratory failure, septic shock, and/or multiple organ dysfunction^5^.

Fatigue was assessed using the 11-item Chalder score^6,7^ with a possible score between 0 and 11(bi-model scoring system). Participants who had a score ≥ 4 were considered fatigued.

For the assessment of the quality of life (QoL), EQ5D5L was used^8,9^, which is the most popular generic preference-based instrument to measure QoL and has the greatest number of country-specific valuations reported around the world. Mobility status of the patient, self-care, daily routine, pain and discomfort, symptoms of depression/anxiety and quality of life using visual analogue marking their subjective satisfaction of their health on the day 0–100 scaler (ruler) were assessed here. Though depression and anxiety could not be diagnosed with the EQ5D5L questionnaire, it can identify the symptoms of depression and anxiety which strongly correlate with the clinical diagnosis. To keep the patients more comfortable, lengthy questionnaires were avoided. Overall questionnaire and EQ5D5L underwent face and content validity with two physicians and a psychologist.

A cognitive function assessment was initially performed with a six-item cognitive impairment test^10^. If the score was > 8, Montreal Cognitive Assessment (MOCA) was performed. The score was given as 18–25 = mild, 10–17 = moderate and < 10 = severe cognitive impairment. Haemoglobin(Hb) level which causes dyspnoea will depend on several factors in each individual**.** There is no sharp threshold value of Hb below which anaemic patients become dyspnoeic and in this study, we considered threshold Hb for dyspnoea as < 10 g/dL for both genders^11^.

Interpretation of the X-rays was done using the RALE classification system^12^. Each lung was assessed individually and depending on the extent of involvement by consolidation or ground-glass opacity a score of 0 to 4 points was given (0—no involvement; 1—less than 25%; 2—25% to 50%; 3—50% to 75%; 4 more than 75% involvement). The overall score was the sum of points from both lungs.

RSNA consensus statement on the reporting of Covid pneumonia was used to report the HRCT studies^13^. A Total severity score was used to evaluate the extent of the HRCT abnormalities. In this classification system, each of the five lobes of the lungs was evaluated for the presence of inflammatory abnormalities, including the presence of ground-glass opacities, mixed ground-glass opacities, or consolidation. Each lobe could be awarded 0 to 4 points, depending on the percentage of the involved lobe: 0 (0%), 1 (1–25%), 2 (26–50%), 3 (51–75%), or 4 (76–100%).

All echocardiograms were performed using the *Philip Epic* echo machine by a cardiologist. Systolic dysfunction defined as left ventricular ejection fraction (EF) < 50% calculated by M-mode (anteroposterior), Simpson’s method and eyeballing and diastolic dysfunction was calculated using the mitral inflow pulse wave doppler, medial and lateral wall LV strain by e’, LA diameter or volume, tricuspid regurgitation velocity supported by the pulmonary venous flow.

### Statistical analysis

The distribution of cases was analysed according to the form of the disease (mild, moderate or severe), the gender of the patient, and other associated pathologies.

Descriptive and inferential statistics were used in the statistical analysis. The range, mean, and standard deviation (SD) for the continuous variables, frequencies, cross-tabulation, and odds ratio (OR) for categorical variables were calculated. Results were expressed descriptively as the number of cases, prevalence (%), and percentage changes for the categories analysed. Descriptive statistics and histograms were derived to observe the distribution of the data, using *t* test, chi-square, and Pearson correlation coefficient as appropriate for analysis.

To summarise the diverse information on frequencies of post-COVID manifestations Cochran's *Q* test in nonparametric tests and regression models were used. The 95% confidence intervals (CIs) were obtained using a random effect model for each clinical manifestation. In addition, patients were evaluated based on their clinical status during acute COVID-19. All presented *p* values were two-tailed, and *p* values < 0.05 were considered statistically significant.

All statistical data were analysed using IBM SPSS Version 21 statistics software in parallel with the Microsoft Excel software.

## Results

One hundred and fifty-nine individuals were treated for COVID infection in UHKDU in July 2021 met the inclusion criteria and 153 agreed to participate in the study. Ninety-two (60.2%) had severe disease, 43 (28.1%) had moderate disease and 18 (11.7%) had mild disease. The mean age was 57.2 (± 16.3) years and 83 (54.2%) were males. Patients were admitted mainly with cough, fever, arthralgia, and headache (Table 1).

**Table 1:** Symptom at the time of admission and characteristics of the participants.

Sociodemography		
Older adults (> 60 years)	75 (49%)	
Younger adults (< 60 years)	78 (51%)	
Marital status	Married 127 (83%)	
	Unmarried 24 (15.7%)	
	Widow 2 (1.3%)	
Occupation	Retired 21 (13.7%)	
	Housewife 55 (35.9%)	
	Own business 28 (18.3%)	
	Employed in government or private 49 (32%)	
Monthly income (LKR)	< 5000—5 (3.3%)	
	5000–20,000—25 (16.3%)	
	20,000–50,000 55 (35.9%)	
	> 50,000 65 (42.5%)	
Education	University 52 (34%)	
	Secondary 77 (50.3%)	
	Grade 8 16 (10.4%)	
	Grade 2 7 (4.6%)	
	Not being to school 1 (0.6%)	
Smoking	20 (13%)	
Alcohol consumption	16 (10.5%)	
Vaccination (at least a single vaccine)	116 (75.8%)	
Fully vaccinated	93 (60.6%) AstraZeneca	5
	Sinopharm	37
	Not specified	51
Co-morbidities	Diabetes	87 (56.9%)
	Hypertension	81 (52.9%)
	Dyslipidaemia	44 (28.7%)
	Ischaemic heart disease	17 (11.1%)
	Asthma/chronic obstructive pulmonary disease	8(5.2%)
Admitting symptoms	Number of symptoms (%)	
Cough	97 (63.4%)	
Arthralgia	97 (63.4%)	
Fever	95 (62.1%)	
Myalgia	77 (50.3%)	
Headache	76 (49.7%)	
Diarrhoea	61 (39.9%)	
Sore throat	60 (39.2%)	
Dyspnoea	51 (33.3%)	
Anosmia	28 (18.3%)	
Rhinorrhoea	27 (17.6%)	
Ageusia	16 (10.5%)	
Vomiting	6 (3.9%)	
Disease severity	Mild	18 (11.7%)
	Moderate	43 (28.1%)
	Severe	92 (60.2%)

All the patients stayed a minimum of 14 days from symptom onset or more in the hospital due to the discharge policy of the government and their disease status.

At least one post covid symptom was reported by all the participants in the second week following discharge (4 weeks after the first symptom). At least one post-COVID symptom was reported by 119 (77.3%) and 92 (60.1%) at 6 weeks and 12 weeks respectively. It reduced to 54 (35.3%) in 6 months then to 25 (15.7%) in 1 year. Five (3%) patients lost to follow-up in 1 year. Only two patients lost their occupation in 2 weeks. The rest of the patient’s occupations were secured and they managed to work after a reasonable medical leave approved by their employers.

All the patients had some form of comorbidity (Table 1). Disease severity is significantly higher in people with diabetes (*p* = 0.004) and hypertension (*p* = 0.005), but not with dyslipidaemia and ischemic heart disease. There was an association between post-COIVD symptoms of dyspnoea and patients with diabetes {0.006(OR 2.68)} and asthma {0.057(OR 0.22)} but not with hypertension.

### Post- COVID symptoms

Most reported post covid symptoms were dyspnoea, arthralgia and insomnia in 2 weeks of discharge and dyspnoea and arthralgia remain the commonest in 6 weeks and 12 weeks (Table 2). There was a statistically significant difference in the persistence of arthralgia (*p* < 0.001) dyspnoea (*p* = 0.015), unsteadiness (*p* < 0.001), cough (*p* < 0.001) and increased frequency of urination (*p* = 0.022) during the followup throughout 3 months period in comparison to the other symptoms as demonstrated by Cochran's *Q* test in nonparametric tests.

**Table 2:** Post covid symptoms.

Symptom n = 153	Two weeks symptomatic (n = 153)	Six weeks symptomatic (n = 119)	3 months symptomatic (n = 92)	6 months symptomatic (n = 54)	1 year symptomatic (n = 25)
Dyspnoea	85 (55.6%)	65 (45.5%)	33 (21.6%)	0	0
Arthralgia	82 (53.6%)	76 (49.7%)	66 (43.1%)	11 (7.8%)	6 (3.9%)
Insomnia	78 (51%)	38 (24.8%)	37 (24.2%)	36 (23.5%)	10 (6.5%)
Weight loss	76 (49.7%)	4 (2.6%)	12 (7.8%)	0	
Unsteadiness	65 (42.5%)	52 (33.3%)	31 (20.3%)	7 (4.6%)	
Sore throat	56 (36.6%)	27 (17.6%)	20 (13%)	1 (0.7%)	
Cough	56 (36.6%)	29 (19%)	30 (19.6%)	4 (2.6%)	
Headache	49 (32%)	49 (32%)	21 (13.7%)	12 (7.8%)	15 (9.8%)
Voice change	42 (27.2%)	0	0	0	
Palpitations	41 (26.8%)	14 (9.2%)	0	0	
Anorexia	37 (24.2%)	3 (2%)	0	0	
Myalgia	36 (23.5%)	24 (15.7%)	9 (5.8%)	1 (0.7%)	
Ageusia	35 (22.9%)	1 (0.7%)	1 (0.7%)	0	
Postural dizziness	33 (21.6%)	13 (8.5%)	23 (15%)	1 (0.7%)	
Increased urinary frequency	32 (20.9%)	15 (9.8%)	17 (11.1%)	6 (3.9%)	
Restlessness	32 (20.9%)	22 (14.4%)	16 (10.5%)	0	
Chest pain	32 (20.3%)	22 (14.4%)	21 (13.7%)	0	
Rhinorrhoea	31 (20.3%)	0	0	0	
Forgetfulness	27 (17.6%)	25 (16.3%	15 (9.8%)	10 (6.5%)	
Calf pain	26 (14%)	14 (9.2%)	14 (9.2%)	0	
Visual impairment	24 (15.7%)	1 (0.7%)	20 (13%)	0	
Aggressiveness	20 (13.1%)	28 (18.3%)	1 (0.7%)	0	
Diarrhoea	2 (1.3%)	3 (2%)	0	0	
Fever	2 (1.3%)	4 (2.6%)	0	0	
Reduced urine output	16 (10.5%)	3 (2%)	0	0	
Paraesthesia	16 (10.5%)	17 (11.1%)	3 (2%)	1 (0.7%)	
Anosmia	1 (0.7%)	0	0	0	
Seizures	1 (0.7%)	0	0	0	

Post-COVID symptoms were significantly associated with disease severity (*p* = 0.004). Dyspnoea {OR 1.136 (CI 95% 0.525–2.455)}, arthralgia {OR 1.83 (CI 95% 0.96–3.503)} and unsteadiness {OR 1.34 (CI 95% 0.607–2.957)} are strongly associated in patients above the age of 60 years. Both genders were equally affected. Increased frequency of urination did not associate with diabetes, high fasting blood sugar or high HbA1c (> 6). Disease severity has not been affected by vaccination status. But post-COVID symptoms were significantly less in the vaccination group (*p* < 0.001).

Overall, 34 (21.4%), 60 (41.5%), 99 (62.3%) and 128 (80.5%) completely recovered in 6 weeks, 12 weeks, 6 months and 1 year respectively.

### Dyspnoea

There were 9 (5.9%) who were anaemic but had no association with dyspnoea. In 2 weeks, 11 (7.2%) had no involvement and 142 (92.8%) people had < 25% involvement in their chest x-rays. None had worse findings. As they become less symptomatic and have no clinical signs, chest x-rays were not performed in subsequent visits. There was no association between chest x-ray findings, HRCT findings, lung functions or echocardiogram findings and symptoms of post-COVID dyspnoea.

### Cardiac functions

Troponin I was performed on 56 patients on admission to the hospital, where only 6(3.9%) got positive results. And they were treated with enoxaparin. Troponin I was only performed in patients with chest pain and patients with ECG changes suggestive of ischemia and myocarditis. There is no association between troponin I and DD nor reduced systolic function (Table 3).

**Table 3:** Respiratory, cardiac and cognitive tests.

	2 weeks (n = 153)				6 weeks (n = 119)				3 months (n = 92)			
Respiratory system												
Chest x-ray	< 25%		Normal									
	142 (92.8%)		11 (7.2%)									
HRCT chest	< 5/25	10–14/25	15–19/25		< 5/25	5–14/25	15–19/25					
	7	3	2		1	4	1					
Lung function test (n = 25) FEV1/FVC									< 70%	70–75%	> 75%	
									7	1	17	
Cardiac functions—echocardiogram (on admission troponin positive in 6, negative in 50)												
Ejection fraction	50–60%		< 50%		< 60%		< 50%		< 60%		< 50%	
	6 (3.9%)		14 (9.2%)		5 (3.3%)		10 (6.5%)		5 (3.3%)		12 (7.8%)	
Diastolic dysfunction	n = 110(72%)				n = 101(65.3%)				n = 64(41.8%)			
	DD1	DD2	DD3	DD4	DD1	DD2	DD3		DD1	DD2	DD3	
	51 (33.3%)	56 (36.6% )	2 (1%)	1 (0.7%)	55 (35.9%)	45 (29.4%)	3 (2%)		39 (25.5%)	24 (15.7%)	1 (0.7%)	
	DM	No DM	< 60 years	> 60 years	DM	No DM	< 60 years	> 60 years	DM	No DM	< 60	> 60
	70	40	47	63	52	18	39	62	45	19	25	39
Cognitive impairment												
MMSE	n = 26				n = 9				n = 3			
	Mild	Moderate	Severe		Mild	Moderate	Severe		Mild	Moderate	Severe	
	16	8	2		7	1	1		1	1	1	
Fatigue	139 (90.3%)				97 (63.4%)				66 (41.1%)			
EQ5DSL-quality of life												
Symptoms of Depression/Anxiety	49 (32%)				30 (19.6%)				8 (5.2%)			
Mobility	27 (17.6%)				25 (16.3%)				4 (2.6%)			
Self-care	9 (5.9%)				5 (3.3%)				2 (1.3%)			
Usual activities	40 (26.1%)				20 (13.1%)				3 (1.9%)			
Pain /discomfort	32 (20.9%)				20 (13.1%)				6 (3.9%)			
Low QoL	60 (39.2%)				36 (23.5%)				11 (7.2%)			
Health today < 50%	12 (7.8%)				3 (2%)				1 (0.8%)			

A significant number {70/87 (80.5%)} of people with diabetes in the group had DD in 2 weeks compared to people without diabetes {40/66 (60.6%)} (*p* = 0.041) and this was reduced to 45/87(51.7%) in 12 weeks when compared with people without diabetes group 19/66 (28.8%) (*p* = 0.05).

There was a significant difference in the incidence {63/75(84%)} of elderly (≥ 60 years) with DD in the group in 2 weeks compared to young (< 60 years) {47/78(60%)} (*p* = 0.001) and this was reduced to 39/75(52%) in 12 weeks when compared with young 25/78(32%) (*p* = 0.012). In 12 weeks, 46 (41.8%) patients remained with the same grade of DD or worsened (*p* < 0.001). Only 18(16.4%) revert to the less severe category.

### Fatigue

Fatigue was significantly associated with arthralgia in 2 weeks (*p* = 0.01), 6 weeks (*p* = 0.003) and 12 weeks (*p* < 0.001). It was associated with dyspnoea (*p* = 0.002) unsteadiness (*p* < 0.001), headache (*p* < 0.001) and insomnia (*p* < 0.001) in 6 weeks. But only headache remained to be significantly associated with fatigue in 12 weeks (*p* = 0.031). Males had more significant (*p* = 0.023) fatigue than women in 12 weeks. The severity of fatigue did not correlate with cardiovascular symptoms, echocardiographic findings, or biomarkers. Restrictive lung patterns or HRCT findings were not associated with fatigue.

### Symptoms of depression or anxiety

In multivariable logistic regression, fatigue and symptoms of anxiety/depression were associated with QoL (*p* = 0.014, *p* < 0.001). There is no association with educational level, gender, or income status.

### Cognitive impairment

Cognitive impairment was found in 26 (female 13, male 13) in a six-item cognitive test in 2 weeks and they were retested with MOCA. There is no difference in gender or age and no association with fatigue (Table 3) They have almost come back to normal senses during 3 months.

### Health-related quality of life

The overall quality of health in 2 weeks was significantly associated with arthralgia (*p* = 0.001), dyspnoea (*p* = 0.013), and unsteadiness (*p* = 0.005) but not with headache (*p* = 0.194). In 6 weeks, 3 months, and 6 months, none of the symptoms were associated with overall health. Over the 6 months, their health improved, and they were back to normal life. In multivariable logistic regression, fatigue and symptoms of anxiety/depression were associated with QoL (*p* = 0.014, *p* < 0.001).

## Discussion

As a single centre-based study, we have observed considerable symptoms occurring at 2 weeks, 6 weeks and 12 weeks after COVID-19 infection affecting physical and mental including the quality of life. Fatigue, dyspnoea and arthralgia have become the most prominent physical symptoms in our study. We have further analysed the association of cardiac function, symptoms of anxiety and depression as well as QoL of everyone with these prominent symptoms.

A similar study in South Africa reported symptoms at 1 month were fatigue or malaise, dyspnoea, headache, weakness of the arms or legs, and confusion or lack of concentration^14^. In a study done in Italy, a high proportion of individuals still reported fatigue (53.1%), dyspnoea (43.4%), joint pain, (27.3%) and chest pain (21.7%) which is compatible with our findings^15^. Though in 3 months, QoL improved in our cohort, it did not come back in the third month after discharge in Chinese studies^16^.

### Cardiac function and dyspnoea

We had 9.2%9 (n = 14) with systolic dysfunction which remained in 7.8% of patients (n = 12) after 12 weeks follow up. This is very much similar to another study done by Tudoran et al.^17^, which showed LV systolic dysfunction in 8.8% of patients at 6–10 weeks follow-up. Unfortunately, they have not assessed the progression or the resolution of such parameters with time. Our study showed that 85.7% of the patient who had systolic dysfunction did not recover even after 12 weeks, which probably would have been due to permanent damage by either by myocarditis or Covid related myocardial ischaemia during the acute illness^18^.

However, the diastolic dysfunction behaved differently compared to the systolic function. This showed some degree of diastolic dysfunction (Grade I-III) was evident in 72% (n = 110) in 2 weeks which reduced to 41.8% (n = 64) by 12 weeks. Tudoran’s study recorded only 16.8% diastolic dysfunction probably because the study population was younger than ours which included patients less than 55 years^17^. Comparatively our study included patients from 18 to 92 years. The resolution of diastolic dysfunction was about 41.8% which was statically significant against the resolution of systolic dysfunction over 12 weeks {2/14 (14.3%) vs 46/110(41.8%)} (*p* = 0.046).

Even though the diastolic dysfunction happened significantly more in the people with diabetes group, evidence of resolution was not different in both arms statistically. However, the *p* value was marginal and the resolution of diastolic dysfunction in the people without diabetes group was more, numerically. In this study, we expect the people without diabetes group to perform well in resolution as the diastolic dysfunction in diabetes is unlikely to resolve further unless solely induced by the Covid infection.

As expected, there was a significant difference in the incidence of diastolic dysfunction in the elderly subgroup at 2 weeks compared to young.

Age is an independent risk factor for diastolic dysfunction, no doubt^19,20^. At 2 weeks of follow-up, we assumed the difference to be due to the age-related diastolic dysfunction confounding the results but interestingly the diastolic dysfunction reduced to 39/75 (52%) by 32% in 12 weeks in the elderly group. In the young group 25/78 (32%) it was only 28%. The decline in diastolic dysfunction in the elderly group was significantly more than in the young group (*p* = 0.012). Hence, we concluded that even in the elderly population, a significant proportion of the diastolic dysfunction was either induced or aggravated by the COVID infection, which would not have settled in 12 weeks, had it been purely to age itself.

In the first systematic echocardiographic examination of 100 consecutive patients requiring hospitalization due to COVID-19 infection, LV diastolic dysfunction (16%) and LV systolic dysfunction (10%)^21^. Our findings of DD are much higher than their population.

### Depression and anxiety

Symptoms of anxiety/depression were found in 49 (32%), 30 (19.6%) and eight (5.2%) at 2 weeks, 6 weeks and 12 weeks respectively in this study cohort. We were unable to diagnose the disease itself than the symptoms with our questionnaire. Anxiety and depression have been identified as the commonest mental health issues followed by COVID-19 in other studies as well^22,23^. This trend of lowering rates of depression and anxiety with time has been observed in several studies^22,24^. However, the prevalence of symptoms of depression and anxiety after 12 weeks is comparatively lower in this study cohort than in the findings of the scoping review by Oliver et al.^24^ which reports the frequency of depressive symptoms in 12 weeks ranged from 11 to 28%. According to their review, the frequency of clinically significant depression and/or severe depressive symptoms ranged from 3 to 12% after 12 weeks^22^.

A large meta-analysis conducted on the South Asian region with a pooled sample of 28,877 revealed that depression and anxiety were higher at 41.3% in the general population based on 35 studies conducted in the region. Another study conducted in Nepal also revealed that depression and anxiety were high among Nepal residents during the pandemic period^23,25^. Hence, depression and anxiety could be a result of the pandemic and its impact on the general lifestyle of people rather than a consequence of the virus. This has to be further analyse with a comparison of the clinical and non-clinical samples.

### Cognitive impairment

Most of the patients assessed for cognitive impairment had difficulties with concentration, memory, receptive language and/or executive function. The same characteristics have been observed worldwide^26^. However, cognitive impairment cannot be concluded without having clear evidence for the premorbid intelligence and ability to respond to a systematic cognitive test in this nature. This study did not have any access to their premorbid intelligence levels. They should act as a clarion call for further research with longitudinal and neuroimaging cohorts to plot recovery trajectories and identify the biological basis of cognitive deficits in post covid patients^27^. A systemic review conducted by Ceban et al.^28^ which included 43 studies on cognitive impairment yielded a significant proportion of individuals who experience persistent fatigue and/or cognitive impairment following the resolution of acute COVID-19. But in our cohort, they have almost recovered in 3 months.

### Strengths and limitations

The main strength of our study is the holistic assessment of the patients by comprehensively assessing the symptoms, psychological health and QoL as well correlation with imaging and laboratory studies.

One of the limitations of our study was the lack of information on the baseline left ventricular functions of the study participants and a lack of premorbid cognitive status. Since the data is from a single centre, it might not be possible to generalise the evidence to other settings. Another limitation is the exclusion of people with mild disease not having any co-morbidities. At the time of the study, people with mild disease not having co-morbidities were self-isolating and did not present to healthcare as per the policy in the country. As a result, these patients were not captured in this hospital-based study. Therefore, these results would not apply to such patients with mild disease free of any co-morbidities.

## Conclusion

In the study population of people with moderate to severe disease or mild disease with co-morbidities, dyspnoea, arthralgia, unsteadiness, cough, chest pain and increased frequency of urination were significantly present throughout 12 weeks period. The severity of the disease and age above 60 years are risk factors for the post-COVID syndrome. Vaccination reduced the post-COVID symptoms.

The reversible and some of the non-reversible diastolic dysfunction in both diabetic and non-diabetic patients and the elderly and young could be associated with COVID infection.

Fatigue was common and arthralgia was associated with it. Symptoms of depression/anxiety associated with QoL though there was a denial. QoL and cognitive impairment improved in 12 weeks. This may indicate that at least 12 weeks need to decide true dementia in post-COVID patients.

## Data Availability

Raw data will be available with the principal investigator (Priyamali Jayasekera, priyamja@yahoo.com/priyamja@kdu.ac.lk) on request.
